# Cholesterol and Oxidative Stress in U.S. Pregnant Women Exposed to Lead

**DOI:** 10.3390/medsci7030042

**Published:** 2019-03-12

**Authors:** Emmanuel Obeng-Gyasi

**Affiliations:** Department of Built Environment, North Carolina Agricultural & Technical State University, Greensboro, NC 27411, USA; eobenggyasi@ncat.edu; Tel.: +1-336-285-3132

**Keywords:** lead pregnancy, pregnancy blood pressure, lead oxidative stress, lead cholesterol

## Abstract

Lead exposure among pregnant U.S. women was examined via the National Health and Nutrition Examination Survey (NHANES) 2009–2016 data to examine its role in bad cholesterol and oxidative stress. Mean values of the clinical markers non-high density lipoprotein cholesterol (non-HDL-c) and gamma-glutamyl transferase (GGT), a marker of oxidative stress, were explored. In four quartiles of lead exposure, clinical makers were compared. Binary logistic regression predicted the likelihood of elevated clinical markers in pregnant compared to non-pregnant women, while linear regression was used to examine associations between blood lead levels (BLL) and the clinical markers of interest. Mean non-HDL-c was statistically significantly more elevated in pregnant women than non-pregnant women. Mean GGT levels were more statistically significantly elevated in the highest quartile of BLL exposure among pregnant women than in the lower quartiles. In binary logistic regression models, pregnant women were statistically significantly more likely to have elevated non-HDL-c, while in linear regression BLL was statistically significantly associated with GGT levels in pregnant women. Lead exposure in pregnant women is an issue of public health concern that must continue to be studied.

## 1. Introduction

Lead is a biologically persistent toxicant that can alter the health status of exposed individuals throughout their lifetime [1]. Exposure may begin as early as pregnancy, with maternal lead serving as an endogenous source of exposure to the developing fetus via maternal bone and blood. Lead can cross the placental barrier and enter the fetal blood circulation. This is of concern as under normal circumstances, between 10 to 15% of ingested lead is absorbed in adults with that number being increased in pregnant women [2].

Women who have been exposed to lead in the past can expose the developing fetus to lead poisoning via cord blood during pregnancy and after birth through breast milk [3,4]. Lead poisoning through breast milk especially is a significant concern if the mother has high lead levels in her system. Lozoff and co-authors, in a study to determine whether breastfeeding for a long period was associated with higher infant lead concentrations, analyzed data from three studies in Costa Rica, Chile, and Detroit; the results demonstrated that when breastfeeding is the sole milk source, there is a correlation between total breastfeeding and infant blood lead levels (BLL) [5]. Namihira and co-authors looked at lead transfer into human milk in Mexican women with an average blood lead of 45 µg/dL. The average lead level in mother’s milk was 2.47 µg/ 100 cc, equivalent to an average intake in an infant of 8.1 µg/kg/day. The daily permissible level by the World Health Organization (WHO) is 5.0 µg/kg/day [6]. Infants are at a higher risk for lead toxicity, as they absorb lead at a higher rate than older individuals do, with absorption reaching up to 50%.

Much of the toxicity associated with lead is related to the fact that it competes with calcium and can affect fetal and maternal bone metabolism [7]. Lagerkvist and co-authors, in a study of an industrial area with a lead-emitting smelter, demonstrated that there was an inverse relationship between BLL and maternal serum calcium levels, with increased lead associated with decreased calcium throughout pregnancy owing to higher lead absorption occurring in the gastrointestinal (GI) tract due to calcium depletion [8].

Research has demonstrated that over 99 percent of lead in whole blood is bound to red blood cells and consequently not capable of crossing the placenta, with the 1 percent of lead in the plasma portion of blood being important concerning fetal exposure [8]. The lead in the plasma portion then crosses the cell membranes and induces health problems commonly associated with lead exposure [9]. After crossing the placental cell membranes via passive diffusion, a direct correlation exists between maternal blood lead levels and fetal blood lead levels [8].

Hertz-Picciotto and co-authors, in a study of 195 pregnant women, found that maternal BLL during pregnancy followed a U-Shaped pattern. Specifically, the authors demonstrated via their study that during late pregnancy, there was a steep increase in lead, especially in women with low dietary calcium consumption [10]. What the above studies confirm is that maternal lead can serve as an endogenous source of infant lead exposure.

Studies now suggest that non-high density lipoprotein cholesterol (non-HDL-c) is a superior predictor of heart disease risk compared to low-density lipoprotein (LDL) cholesterol [11]. Non-HDL cholesterol, a reflection of LDL cholesterol and very low-density lipoprotein (VLDL) cholesterol, is the difference between HDL cholesterol and total cholesterol and is a good marker to measure the bad cholesterol in individuals. Lead has been shown to be associated with adverse cholesterol levels in occupational settings and in the U.S. general adult population [12,13]. 

Oxidative stress is the mechanism by which much of lead-induced pathology comes about [14]. In this study, Gamma-glutamyl transferase (GGT) is the marker of oxidative stress, as it can serve as a sensitive enzyme for oxidative stress [15,16]. Oxidative stress plays a role in cholesterol accumulation via impairing the expression of receptors involved in cholesterol flux in macrophages [17]. 

This novel study sought to understand lead exposure levels and clinical markers among a sample of pregnant U.S. women, as lead is still an issue of public health significance. 

## 2. Materials and Methods

### 2.1. Study Hypothesis

The hypothesis of this study was that lead exposure in pregnant women is associated with oxidative stress and bad cholesterol owing to internal exposure due to metabolic mechanisms arising from pregnancy, such as mobilization of lead from bones. The objectives of this study were to investigate the effects of lead exposure by analyzing non-HDL-c and GGT in pregnant U.S. women while adjusting for age and body mass index (BMI). 

### 2.2. Research Design

The relationship between lead and GGT and non-HDL-C, which was calculated by subtracting total cholesterol from HDL cholesterol, was explored with NHANES 2009–2016, which is a representative sample of the U.S. noninstitutionalized population. Data were analyzed for pregnant and non-pregnant women. The 2009–2016 datasets were put together using the publically available NHANES web tutorial [18]. 

The Institutional Review Board (IRB) approval for NHANES was been obtained by the National Center for Health Statistics (NCHS) with adult participants providing written informed consent directly [19].

Whole blood samples were used to conduct metal assays in the NHANES 2009–2016. Metal assays in blood samples were conducted at the Division of Laboratory Sciences within the National Center for Environmental Health (NCEH) at the Centers for Disease Control and Prevention (CDC) (Atlanta, GA, USA). Inductively coupled plasma mass spectrometry (ICP-MS; CDC method No. ITB0001A) measured BLL with 0.07 µg/L being the lower limit. A Beckman Synchron LX20, Beckman UniCel® DxC800 Synchron was used to measure GGT levels (Collaborative Laboratory Services) including the Roche Modular P chemistry analyzer (University of Minnesota, Minneapolis, USA). Stata SE/15.0 (StataCorp, College Station, TX, USA) was used to adjust for the sample weights, strata, and clusters of the complex design.

### 2.3. Statistical and Analytical Approaches

Data from adults indicating if they were pregnant or not were analyzed in this cross-sectional study. Quartiles of exposure were created using BLL based on the BLL data distribution within the database. The mean values for the markers of interest were then examined within the quartiles of exposure to assess their values. Binary logistic regression was used to predict the likelihood of increased markers of interest with the binary dependent variable being pregnancy status (yes or no). Linear regression was used to determine association between BLL and clinical markers of interest in pregnant women. Each exposure outcome variable was explored in individual models in both linear and logistic regression models. The data was adjusted for age and BMI, with the complex design and weights adjusted for the analysis of the demographic and quartile data, while for the regression data the design and weights were not factored in due to inadequate data in all strata. The data was not perfectly normally distributed according to the Shapiro-Wilk test, so all data were natural log transformed. A p-value of less than 0.05 determined statistical significance.

## 3. Results

### 3.1. Sociodemographic and Clinical Markers

The sociodemographic and clinical markers of this study are explored in this section. Mean BLL were higher in non-pregnant compared to pregnant women but the difference was not statistically significant. Mean age and Body Mass Index (BMI) were significantly different between pregnant and non-pregnant women. Also, mean GGT was more elevated in non-pregnant as compared to pregnant women, with mean non-HDL-c being significantly more elevated in pregnant women. The sociodemographic and clinical makers can be found in Table 1 below.

### 3.2. Clinical Markers across Quartiles of Exposure

The clinical markers of interest were examined to see how they manifested across different quartiles of exposure in pregnant women. The mean blood lead levels in various quartiles are shown in addition to the mean values of the markers of interest. The range of quartile values are shown below the quartile. Table 2 summarizes the results. 

### 3.3. Clinical Markers in Pregnant Women

Binary logistic regression was performed to see the likelihood of elevated or diminished clinical markers in pregnant women compared to non-pregnant women. The results can be found below in Table 3. 

Linear regression was performed to see the association between BLL (lnBPb) and clinical variables of interest in pregnant women. The results can be found below in Table 4.

## 4. Discussion

In this study, pregnant women were less exposed to lead than non-pregnant women, but the difference was not statistically significant. The equivalent exposure to lead between pregnant and non-pregnant women may be due to the heightened awareness of leads effects on pregnant women’s health in the U.S. 

Lead affects nearly every organ system within the human body [13,20,21,22], with exposure occurring in many environments [23,24,25,26]. Pregnant women absorb a higher percentage of ingested lead than the general population, making their health an issue of public health concern [2]. Lead absorbed during the life course can be mobilized up to 6 months postpartum and has been shown to be higher during lactation than in pregnancy [4]. Also, the fact that lead mimics calcium in several processes demonstrates that it can act as a source of toxicity altering several processes of biological importance [27]. This study demonstrated that U.S. pregnant women are less exposed to lead than non-pregnant women. This is key as lead exposure during pregnancy is highly correlated between mothers and infants. Schell and co-authors, when measuring BLL for 200 mother-infant pairs, found the *Pearson correlation coefficient* of maternal to newborn BLL to be 0.66 in the first trimester, 0.53 in the second trimester, 0.69 in the third trimester, and 0.91 at delivery [28]. Additionally, when breastfeeding is the sole milk source, there is a correlation between total breastfeeding and infant BLL [5]. Even though pregnant women are less exposed to lead than non-pregnant women, no level of lead exposure is safe. 

Lead is potentially involved in the synthesis and attenuation of enzymes that are key to cholesterol synthesis. Lead works to upregulate enzymes involved in the cholesterol biosynthetic process while suppressing cholesterol catabolic enzymes [29]. In this study, pregnant women had higher mean non-HDL cholesterol compared to non-pregnant women. Also, they were more likely to have elevated non-HDL cholesterol compared to non-pregnant women in binary logistic regression. This potentially speaks to leads effects in promoting bad cholesterol formation in pregnant women, but other factors during pregnancy such as changes in sex steroid hormones, in addition to hepatic and adipose metabolism, may contribute to raising these lipids [30].

The pro-oxidant/antioxidant balance in the cells of mammals can be altered by lead poisoning via lead-induced oxidative stress [31]. In this study, pregnant women who were exposed to lead in the highest quartile of exposure had statistically significantly higher oxidative stress compared to those exposed in lower quartiles. The large SE of GGT in Quartile 4 indicates that the result may be partly a reflection few women in this quartile. In linear regression, there was a statistically significant positive association between BLL and GGT in pregnant women, indicating a potential mechanism of disease. In binary logistic regression, pregnant women were statistically significantly less likely to have oxidative stress than non-pregnant women, which may be due to behavioral and environmental exposures of the studied population. 

### Limitations

Owing to leads long half-life in bone, using K-Shell X-Ray Fluorescence (KSXF) would have a better indicator of long-term exposure. As BLL is a measure of shorter-term exposure, using it in addition to bone lead levels would have provided a comprehensive view of the participant’s exposure [32]. 

Another limitation of this study is that it depicts a U.S. population where exposure levels are not as high as they are in other countries; thus, results may not be generalizable to a global population. Finally, this study is cross-sectional; a longitudinal study may have yielded different results.

## 5. Conclusions

Lead exposure in pregnancy is an issue of public health importance. Lead toxicity may further exasperate oxidative stress in pregnant women in addition to promoting non-HDL-c. The burden of lead may be passed on to the developing fetus, which may acquire damage that could affect it during its lifetime. 

## Figures and Tables

**Table 1:** Sociodemographic and clinical markers in the study.

	Pregnant *N* = 256	Not Pregnant *N* = 4611
Mean BLL (95% CI)	0.70 (0.45–0.95)	0.80 (0.76–0.84)
Mean Age *	29.08 (28.23–29.94)	32.24 (31.82–32.66)
Mean BMI *	29.78 (28.73–30.82)	28.57 (28.28–28.84)
GGT	16.05 (11.49–20.61)	19.77 (18.69–20.85)
Non-HDL *	148.03 (139.80–156.27)	125.91 (124.49–127.33)

* *p* < 0.05 Statistically significant difference between pregnant non-pregnant women.

**Table 2:** Mean clinical factors and quartiles of exposure in pregnant women.

	Quartile 1 (0.05–0.58)	Quartile 2 (0.59–0.92)	Quartile 3 (0.93–1.52)	Quartile 4 (1.53 +)
GGT *	15.34 (2.33)	13.37 (1.95)	13.78 (1.37)	94.11 (38.16)
non-HDL-c	148.79 (7.66)	167.94 (8.41)	125.97 (14.00)	135.07 (6.72)

* *p* < 0.05 Statistically significant difference between quartiles 1, 2,3, and 4.

**Table 3:** Binary logistic regression of pregnant women compared to non-pregnant women for clinical variables of interest.

Variables	Adjusted Odds Ratio (95% CI) *	*p* Value
GGT	0.283 (0.206–0.390)	0.0001
non-HDL-c	5.37 (3.30–8.73)	0.0001

* Adjusted for age and BMI.

**Table 4:** Linear regression association between BLL and variables of interest in pregnant women.

Variables	lnBPb (SE) *	*p* value
GGT	0.169 (0.007)	0.020
non-HDL-c	−0.34 (0.129)	0.794

* Adjusted for age and BMI.
